# Age-Friendly Teaching and Learning: New Roles for Older Adults Across the Curriculum

**DOI:** 10.1093/geroni/igaa057.1785

**Published:** 2020-12-16

**Authors:** Kimberly Farah, Joann Montepare

**Affiliations:** Lasell University, Newton, Massachusetts, United States

## Abstract

The pioneering Age-Friendly University (AFU) framework, with its set of ten guiding principles, advocates for enabling older adults to participate fully in educational activities that promote positive and healthy aging. In addition, the AFU principles call attention to bringing younger and older learners together around educational goals, and engaging learners in collaborative classroom experiences that facilitate the reciprocal sharing of expertise between learners of all ages. Implied, but not articulated, in these principles is the idea that older adults’ expertise, skills, and talents can also be tapped to support classroom learning goals and extend teaching strategies. This presentation will show how older adults can serve as valuable educational allies in classrooms across the curriculum with examples of crime scenario developers in a forensics class, conversation partners in an international oral communication class, and professional interviewers in an internship skills class. Evidence will argue that these roles enhance student learning.

